# Evidence of Semantic Processing in Parafoveal Reading: A Rapid Parallel Visual Presentation (Rpvp) Study

**DOI:** 10.3390/brainsci11010028

**Published:** 2020-12-29

**Authors:** Danila Rusich, Lisa S. Arduino, Marika Mauti, Marialuisa Martelli, Silvia Primativo

**Affiliations:** 1 Department of Human Sciences, LUMSA University, P.zza delle Vaschette 101, 00193 Rome, Italy; l.arduino@lumsa.it (L.S.A.); s.primativo@lumsa.it (S.P.); 2 Department of Psychology, Sapienza University, Via dei Marsi, 78, 00185 Rome, Italy; marika.mauti@gmail.com (M.M.); marialuisa.martelli@uniroma1.it (M.M.)

**Keywords:** reading, parafovea, semantic processing, rapid parallel visual presentation paradigm, parafoveal-on-foveal effect

## Abstract

This study explores whether semantic processing in parafoveal reading in the Italian language is modulated by the perceptual and lexical features of stimuli by analyzing the results of the rapid parallel visual presentation (RPVP) paradigm experiment, which simultaneously presented two words, with one in the fovea and one in the parafovea. The words were randomly sampled from a set of semantically related and semantically unrelated pairs. The accuracy and reaction times in reading the words were measured as a function of the stimulus length and written word frequency. Fewer errors were observed in reading parafoveal words when they were semantically related to the foveal ones, and a larger semantic facilitatory effect was observed when the foveal word was highly frequent and the parafoveal word was short. Analysis of the reaction times suggests that the semantic relation between the two words sped up the naming of the foveal word when both words were short and highly frequent. Altogether, these results add further evidence in favor of the semantic processing of words in the parafovea during reading, modulated by the orthographic and lexical features of the stimuli. The results are discussed within the context of the most prominent models of word processing and eye movement controls in reading.

## 1. Introduction

An interesting debate in the literature on reading concerns the nature of the information extracted from words located in the parafovea. It is well known that the foveal region (subtending a visual angle of 2°) is the region with the highest visual acuity, generally comprising 6–8 characters, and that it is clearly distinguished from the parafoveal region (with a visual angle of 2–5°), which extends beyond the foveal region for up to approximately 15–20 characters [1]. Despite a decreased visual acuity, accompanied by reduced attentional resources and visual span [2,3], it has been shown that readers can nonetheless extract some important information from the parafoveal region [4,5]. In the literature, two main effects have been described that can provide information regarding the processing of parafoveal elements: the parafoveal preview effect (PPE) and the parafoveal-on-foveal (PoF) effect.

The PPE is generally tested by using a display change paradigm such as the boundary paradigm [6], in which a target word in a sentence is previewed by an identical or related word (i.e., valid) or a nonword (i.e., invalid condition) until the reader’s eyes cross an invisible boundary positioned immediately before it. A considerable facilitation of eye movement metrics has been observed in the case of valid words, both for sublexical and lexical influences (for a review, see [7]). In addition, the frequency of the parafoveal word has been showed to affect its preview benefits, and highly frequent parafoveal words received shorter first fixations and gaze durations than less frequent ones [8]. Moreover, an increase in gaze duration on the parafoveal word was shown when the foveal word was short and the parafoveal word was less frequent [9].

However, it is still a matter of debate whether, and to what extent, parafoveal information may be deeply processed up to the semantic level. For example, Rayner, Balota and Pollatsek [10] and Rayner, Schotter and Drieghe [11] found a lack of semantic processing of the parafoveal word in sentence reading. In this study, the authors used four alternative preview conditions of the target: the target word itself (e.g., razor), a semantically related word (e.g., blade), a semantically unrelated word (e.g., sweet) and a nonword orthographically similar to the target (e.g., razar). Fixation times measured on the target word showed no evidence of a semantic preview benefit. Indeed, no significant differences were observed between the semantically related and semantically unrelated conditions, with shorter fixation times only observed for the identical condition, evidencing merely a visual orthographic preview benefit. Conversely, by using the same paradigm for sentence reading in the German language, Hohenstein and Kliegl [12] showed that the target word was recognized faster when it was semantically related with the preview. In order to further investigate the extent of the semantic processing in parafoveal vision, Schotter [13] then ran two sentence reading experiments with various related preview conditions, including identical (e.g., curlers and curlers), synonymous (e.g., rollers and curlers) and semantically related (e.g., styling and curlers) conditions, as well as a semantic unrelated control condition (e.g., suffice and curlers). Reading times were observed to be shorter in the synonymous and identical conditions than in the others. Subsequent studies showed the presence of a semantic preview benefit only in the case of constrained sentences [14] and previews that were plausible in the sentences [15], suggesting that, in sentence reading tasks, it is difficult to disambiguate between a pure semantic preprocessing effect and a more general contextual plausibility effect of the preview [15,16,17].

The PoF effect refers to the possibility that parafoveal word processing can influence foveal word processing during reading [9,18,19,20,21,22,23,24,25,26,27,28]. The PoF effect, as much as the PPE, has been mainly studied in the context of sentence reading by using either the boundary paradigm or the moving window paradigm [29], leading to similar limits as those reported above in terms of data interpretation. For example, in a sentence reading task, Inhoff, Radach, Starr and Greenberg [18] varied the semantic relatedness of two adjacent words with three conditions: identical (e.g., mother and mother), semantically related (e.g., mother and father), and semantically unrelated (e.g., mother and garden). They thus found that the gaze duration on the preceding word was significantly shorter when the following word was either identical or semantically related than when it was unrelated.

A larger set of studies using a display change paradigm (such as the boundary paradigm) for sentence reading tasks has, however, failed to report systematic semantic parafoveal-on-foveal effects [30,31,32]. Accordingly, in using highly arousing emotional words, emotionally neutral words and identical words as previews for the target in the parafovea, Hyona and Haikio [32] did not find any differences in gaze duration among parafoveal word semantic preview conditions, suggesting that the semantic component was not identified parafoveally.

Evidence of semantic processing have been shown in German [12,33] and Chinese [34,35,36]. Only a few pieces of evidence of semantic parafoveal preview effects have been reported in English [13,37]. Such a difference among languages might depend on the orthographic depth [13]. In fact, it has been suggested that the semantic representations might be activated later in opaque orthographies (as compared to more transparent ones) and that there is simply not enough time during parafoveal preview for information to feed up to semantics.

More recently, Snell, Declerck and Grainger [38] directly compared the semantic PoF effect in processing isolated words versus sentence-embedded words, and they found a semantic parafoveal-on-foveal effect in categorization tasks using isolated words in a flanking paradigm (Experiments 2 and 3), but not in a sentence reading task (Experiment 1), with the authors claiming the data suggested that sentence constraints lead the reader to spatiotopic sentence level processing, which interferes with semantic processing, thus resulting in the lack of a semantic parafoveal benefit. However, it is also plausible that the semantic categorization task may have strongly elicited semantic processing of the stimuli.

**Figure 1:**
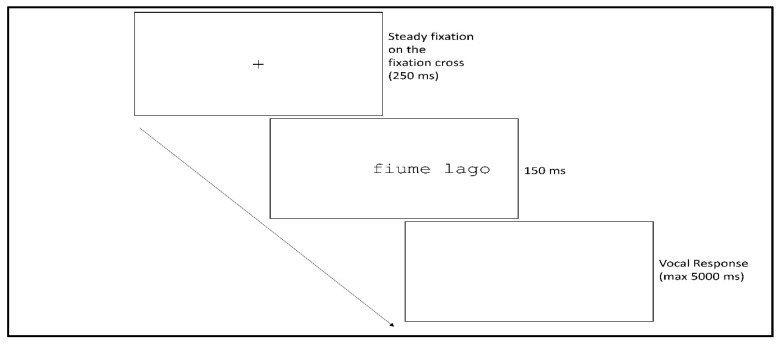
Schematic representation of the rapid parallel visual presentation (RPVP) paradigm.

In this study, we explore parafoveal semantic processing using the rapid parallel visual presentation paradigm (RPVP), whereby two words are briefly and simultaneously presented (Figure 1), with one in the fovea region and one in the parafovea region. There are multiple advantages in using this paradigm. Participants are asked to read both words, and thus the task does not inherently elicit the deeper processing of one over the other. Furthermore, by using pairs of words as stimuli, we can exclude confusion relating to sentence processing. Finally, such a paradigm allows for a strictly controlled experimental procedure, in terms of both stimuli presentation timing and experimental linguistic stimuli manipulation.

Overall, the aim was to investigate the existence of semantic parafoveal processing and, by recording eye movements and vocal reaction times, how it may be modulated by perceptual and lexical variables, such as word length and written word frequency, as well as exploring the preview benefit effect by analyzing the correct naming percentage of parafoveal words and the parafoveal-on-foveal effect by analyzing foveal word reaction times as a function of different parafoveal stimulus features. We thus orthogonally manipulated the stimulus length, written word frequency and the semantic relationship between the two stimuli. We hypothesized that if the parafoveal processing reaches a semantic stage, the parafoveal word should achieve higher accuracy when it is semantically related to the foveal word, as opposed to when the two stimuli are not semantically related. We also hypothesized that the semantic preview effect should be modulated by the length and written frequency of both words, with a higher accuracy attained when both words are short (i.e., both words are within the perceptual span [3,5]) and highly frequent [8,9]. Furthermore, in terms of the PoF effect, we hypothesized shorter foveal word reaction times when the parafoveal word is semantically related. Finally, a qualitative analysis of parafoveal word reading errors further elucidates the contribution of perceptual and semantic components to semantic parafoveal processing.

## 2. Materials and Methods

### 2.1. Participants

Thirty undergraduate students from Libera Università Maria Ss. Assunta (LUMSA) University gave written informed consent for their participation in the study. The average age of the participants was 24 years old (range = 19–31 years, s.d. = 4.2, F:M = 20:10). All participants reported themselves to be non-dyslexic and had normal or corrected-to-normal sight. The participants were naive with regard to the final purpose of the experiment.

### 2.2. Stimuli

One hundred twenty-eight pairs of words, selected from the LEXVAR database (Barca, Burani and Arduino, 2002 [39]), were used as stimuli. The word length in letters (mean = 6.2, range = 4–9, s.d. = 1.7), adult written word frequency (mean = 133.4, range = 1–2253, s.d. = 252.9) and semantic relationship between the two words were manipulated orthogonally. The stimuli were thus short (4–5 letters) and long (7–9 letters) and of low (1–20 occurrences per million words) and high (>51 occurrences per million words) adult written word frequencies, using a frequency count of 1,500,000 words drawn from written Italian texts (Istituto di Linguistica Computazionale, 1989), whether they were semantically related or not.

Two lists were created, one semantically related (SR) and one semantically unrelated (SU), each consisting of 64 pairs of words. The two lists of words were matched by age of acquisition, familiarity, imaginability, concreteness, adult written frequency, number of orthographic neighbors, bigram frequency, number of syllables and length in letters (two-tailed t-tests, all *p* > 0.05). Each list was composed of pairs of words labeled w1 for the first word presented in the fovea and w2 for the second word presented in the parafovea. Half of w1 and w2 were short (*N* = 32 pairs) and half were long (*N* = 32 pairs). Half of w1 and w2 were of a high frequency (*N* = 32 pairs) and half were of a low frequency (*N* = 32 pairs). All possible combinations among word lengths and frequencies were used. The lengths in letters and the written word frequencies of the stimuli are reported in Table 1. The semantic relation between words was previously established through the administration of a self-report questionnaire (7-point Lickert scale) to 62 participants (age range = 18–26 years, age mean = 19.4, s.d. = 1.3, F:M = 49:13) who did not take part in the main experiment. Participants were asked to rate on a scale from 1 to 7 the semantic relatedness of the two words. Semantically related words were categorized as being significantly more related (mean = 5.4, s.d. = 0.8, Min–Max = 3.1–6.7) compared to semantically unrelated words (mean = 1.5, s.d. = 0.4, Min–Max = 1.0–2.9), t2(126) = −34.8, *p* < 0.001. Synonyms and antonyms were excluded from the set of stimuli pairs.

### 2.3. Software and Apparatus

The software Experiment Builder (SR Research Ltd., Mississauga, ON, Canada) was used for programming and running the experiment. Monocular eye movements were recorded in binocular vision via an SR Research Ltd. Eye Link 1000 eye-tracker, sampling at 1000 Hz in order to ensure the fixation stability and retinal position of the stimuli prior to their presentation on the screen. Participants were seated at a 60 cm distance from the display, with their heads held firm by the use of a chin rest. Stimuli were presented on a 17 inch LED screen (1366 × 768 px, 60 Hz). Participants’ voices were recorded via a one-way microphone connected to an external sound card (M-track 2 × 2).

### 2.4. Experimental Procedure

A nine-point calibration and validation procedure and a drift correction to ensure fixation stability were performed before each individual trial. Subsequently, a fixation cross (subtending a visual angle of 0.5°) was then presented on the left-hand side of the screen. The onset of the stimuli was then triggered by steady fixation from the participant on the fixation cross which, positioned between the second and the third letters of the foveal word (the optimal viewing position [1]), remained on the screen for a minimum of 250 ms. After the offset of the fixation point, the two words were randomly sampled, without repetition, from the lists of semantically related or semantically unrelated pairs and simultaneously shown for 150 ms on the screen, one in the fovea region (w1) and one in the parafovea region (w2). After the stimulus presentation, a blank screen appeared until the participant’s verbal response (no mask was used). The words were presented in the Courier New font, a font with constant center-to-center letter spacing independent of the letter width. Each letter subtended a visual angle of 0.5°. The spatial extension of the stimuli, extending from 1° left of the fixation cross to the right side, ranged from a visual angle of 4.5° (both words short) to 9.5° (both words long). The task of the participants was to read both words aloud. Accuracy was measured for w1 and w2, and reaction times were measured for w1.

## 3. Results

### 3.1. Accuracy

**Figure 2:**
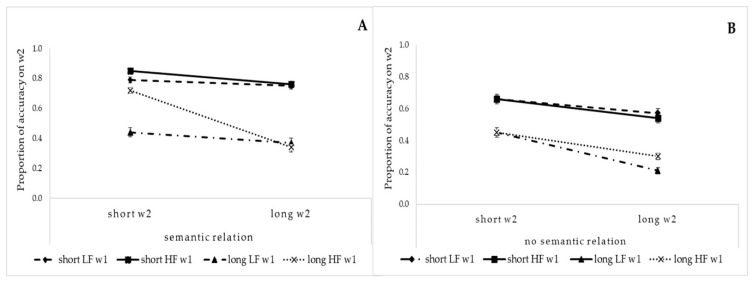
Accuracy on w2 as a function of w1 length and frequency in the semantically related condition (Panel **A**) and in the semantically unrelated condition (Panel **B**). LF = low-frequency; HF = high-frequency.

On average, the accuracy was 100% on w1 and 56% on w2 (range = 25–89%). Thus, no further analysis was performed on the accuracy of w1. In order to investigate the proportion of errors in the different experimental conditions, a logistic regression analysis was run on the binary accuracy data, with participants as repeated measures. The length (short or long), frequency (low or high) and semantic relationship (semantically related or semantically unrelated) between the two words represented the independent variables. A higher accuracy for w2 was observed when w1 was short than when it was long (62.4% vs. 37.6%; χ^2^(1) = 295.9, *p* < 0.0001), when w2 was of a high frequency rather than a low frequency (53.5% vs. 46.5%; χ^2^(1) = 16.8, *p* < 0.0001) and when there was a semantic relation between the two words (57.4% vs 42.6%; χ^2^(1) = 95.5, *p* < 0.0001). Moreover, the three-way interactions of w1 length, w2 frequency and semantic relation (χ^2^(1) = 22.3, *p* < 0.0001) and w2 length, w1 frequency and semantic relation (χ^2^(1) = 12.3, *p* < 0.0005) were statistically significant. Crucially, the four-way interaction of w1 length, w2 length, w1 frequency and semantic relation (χ^2^(1) = 3.8, *p* < 0.05) was statistically significant. As shown in Figure 2 (Panels A and B), the interaction indicated that while a higher accuracy emerged when both words were short rather than long (both *p* < 0.001) for both the semantically related and semantically unrelated words, only in the semantically related condition did the word frequency affect the accuracy. Indeed, in the semantically related condition, when w1 was a long, low-frequency word, the accuracy was low regardless of the length of w2 (*p* < 0.001). Conversely, when w1 was a long, high-frequency word, an increase in accuracy was observed only for a short w2 (*p* < 0.001).

### 3.2. Reaction Times

Reaction times (RT) were measured in milliseconds as the interval between the onset of the two stimuli on the screen and the onset of the vocal response of the participant. The participants named w1 and w2, in this order, and the RT was measured on w1. Only the RTs of trials in which the participant was accurate, concerning both w1 and w2, were included in the analysis. Trials in which the response time was beyond 2.5 standard deviations from the mean were discarded (1.9% of all trials). A factorial ANOVA was run with the reaction times as dependent variables and the length, frequency and semantic relationship between the two words as independent variables.

**Figure 3:**
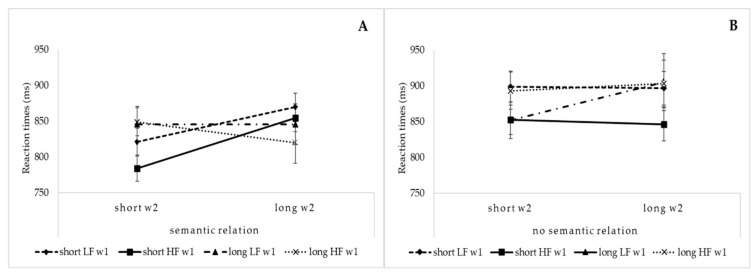
Mean vocal reaction times (ms) when w1 is short and long (Panel **A** = semantically related; Panel **B** = semantically unrelated). LF = low-frequency; HF = high-frequency.

The results showed shorter RTs when the two words were semantically related than when they were not (57.4% vs. 42.6%; F(1,1781) = 12.6, *p* < 0.0005, η_p2_ = 0.007). The significant interaction of the w1 length, w2 length and semantic relation (F(1,1781) = 4.8, *p* < 0.05, η_p2_ = 0.002) indicated that shorter RTs were observed when both words were short, but only when semantically related. Moreover, the significant interaction of the w1 frequency, w2 frequency and semantic relation (F(1,1781) = 3.8, *p* < 0.05, η_p2_ = 0.002) indicated that shorter RTs were observed when both words were of a high frequency, but only when semantically related (see Figure 3, Panel A and B).

In order to investigate whether the performance for w2 (in the previous analysis, only correct w2 responses were included) modulated w1 reaction times, a one-way ANOVA variance analysis was applied, with the reaction times for w1 as the dependent variable and the accuracy of reading w2 (correct, no response and misread) as grouping variables. This revealed significant differences among the conditions (F(2,3173) = 11.7, *p* = 0.00001, η_p2_ = 0.007), indicating significantly shorter reaction times in the case of correct readings of w2 (mean = 851.1, s.d. = 5.9, *p* <  0.001) versus no responses (mean = 896.9, s.d. = 8.3) and misread words (mean = 890.9, s.d. = 11.9, *p* = 0.008). Therefore, the correct identification of the parafoveal word appeared to speed up the RTs measured for w1.

### 3.3. Qualitative Errors Analysis

The total number of w2 reading errors was 1571 (44% of response), with 70.1% (*N* = 1101) of the total being no responses and 29.9% (*N* = 470) being misread words. The distribution of the errors (no responses and misread words) according to the different experimental conditions is represented in Table 2. A Wilcoxon signed-rank test was run in order to compare the differences among experimental conditions for the no responses and misread words. As can be observed from Table 2, a larger number of no responses on the parafoveal words was observed for long and low-frequency words and when there was no semantic relation with the foveal word. A larger number of misread words was observed when w2 was short and when there was no semantic relation with w1. In this case, the written frequency did not modulate the proportion of errors.

We divided the misread errors into three major categories: perceptual, semantic and a combination of the two. The errors were perceptual if (1) it had the first letter in common with the target word (e.g., faro and ferro, meaning lighthouse and iron); (2) it shared important perceptual features, such as a double consonant (e.g., cervello and cavallo, meaning brain and horse); (3) it had the same length in letters (e.g., note and ponte, meaning night and bridge); (4) it consisted of a letter transposition (e.g., sorso and rosso, meaning sip and red); and (5) it consisted of a letter substitution (e.g., rata and rete, meaning instalment and network). The errors were semantic if they were semantically related to the words actually presented (e.g., pioggia and grandine, meaning rain and hail). Finally, a proportion of the errors had both a perceptual and a semantic component (e.g., gobba and gabbia, meaning hump and cage). The misread errors (*N* = 470) were therefore found to be distributed as follows: 64% perceptual (*N* = 302), 3.4% semantic (*N* = 16) and 26.8% a combination of both (*N* = 126). The remaining 26 errors (5.5%) were other types of errors not falling the three categories (e.g., pipa and aperto, meaning pipe and open).

Overall, the majority of misread words appear to have been driven by a misperception of the parafoveal word at the level of letter encoding. It is possible that when the semantic processing of the parafoveal word was not achieved, only lower level perceptual processing was accomplished, leading to the identification of some but not all perceptual elements.

## 4. Discussion

In this study, we investigated the semantic processing of Italian words in parafoveal vision during the speed reading of pairs of words and its interactions with both the orthographic and lexical properties of stimuli. Accordingly, we orthogonally manipulated the stimuli word length, written word frequency and semantic relatedness between the two words. Pairs of semantically related and semantically unrelated words were briefly and simultaneously presented on the screen, with one in the fovea region and one in the parafovea region. The participants’ task was to read both words aloud. The accuracy for the parafoveal word and reaction times for the foveal words were measured.

The results from the analysis of accuracy revealed that participants were more accurate in reading the parafoveal word when there was a semantic relation between the two words than when there was not. Moreover, the semantic effect was strengthened by lexical and perceptual components. Indeed, greater accuracy in reading the parafoveal word was observed with semantically related words when the foveal word was short and highly frequent and the parafoveal word was short. The measured reaction times revealed the presence of a semantic parafoveal-on-foveal effect (i.e., the influence of features of the parafoveal word on the processing of the word in the fovea). Indeed, the presence of a semantic relation between the two linguistic stimuli was observed to speed up the naming of the foveal word when both words were short and highly frequent.

Altogether, these results provide evidence in favor of the semantic processing of words in the parafovea region during reading [12,13,14,15,40]. Our data are partially in accordance with both the parallel and serial models of reading and, in the following, we make an attempt to explain the results within both frameworks.

The parallel model of word recognition features the simultaneous processing of not only perceptual and lexical information, but also high-level semantic information from parafoveal vision, as in the OB1-Reader model [41]. The model thus proposes higher-order (semantic and syntactic levels) parallel processing across multiple words, integrating the main assumptions of the relative position coding for word recognition model [42] and of the parallel attentional gradient allocation of the SWIFT model [43]. Indeed, it first assumes parallel letter identification in multi-letter strings and secondly a parallel processing of multiple words through a graded distribution of visuo-spatial attention. According to this model, orthographic word identities are processed in parallel, with each word identity location-specific in the sentence. This mechanism allows parallel independent activation of semantic and syntactic processing by multiple words, which then feeds information into higher-level sentence comprehension processes. In the case of paradigms that do not engage in sentence-level representations, the connectionist perspective adopted by the model accounts for the simultaneous processing of multiple words, based on a spreading activation from the word form to higher-order (semantic and syntactic) features. In this interactive framework, the parafoveal-on-foveal effect seems to be evidence of the parallel and simultaneous processing of words during reading up to the semantic level [38,44,45,46].

However, the large amount of errors in naming the parafoveal word is not fully justified by parallel models, pushing toward a serial model of word processing. In fact, our results are also in accordance with the hybrid mechanism of the saccade triggering model [47], based on the serial assumption of attention allocation in reading (e.g., the E-Z Reader model [48]). In this model, word processing consists of two subsequent steps: the familiarity check that triggers saccades and the completion of lexical access. Accordingly, the theory goes that readers plan saccades before full processing of the word, and that parafoveal information can facilitate processing during reading via trans-saccadic integration (short fixations on a target word when the preview is more similar to it) or forced fixations (short fixations on words that would be skipped independent of the target). Indeed, the majority of errors on the parafoveal word and their perceptual (rather than semantic) nature may suggest that, although the semantic processing is initiated in parafoveal vision, it is not completed. It is thus possible that a full, deep processing of the word is only attainable once the word is fixated, as suggested by the serial models of eye movement controls in reading. However, the existence and types of parafoveal influence during word processing in reading merely underlies a broader open debate between the serial eye movement control (e.g., E-Z Reader) model and the parallel (SWIFT) model. In this framework, our data cannot distinguish between the two models. Furthermore, recently Schotter et al. [37] demonstrated that a serial attention model of eye movement control in reading, such as the E-Z Reader, is not necessarily inconsistent with some processing of the semantic information in the parafovea region. Indeed, throughout model simulations, the authors demonstrated that the information obtained from the parafoveal word, although imprecise, could be also used to initiate higher level processing within a serial framework of word processing.

While the present study’s qualitative error analysis suggests a strong influence from low-level perceptual stimuli features, the results on reading speed provide strong evidence in favor of possible semantic processing in the parafovea region, highlighting the presence of both a semantic parafoveal preview benefit and a semantic parafoveal-on-foveal effect during reading that demands further investigation. Indeed, this study indicates that a limited capacity mechanism that decodes only partial information might be sufficient to trigger high-level semantic word processing.

Despite the already mentioned advantages of the RPVP paradigm, as also clearly showed by Snell et al. [38], one limitation of our study is that it does not reflect natural reading and that specific attentional strategies might be elicited. Unlike vocal RTs, the paradigm used enables one to assess the parallel processing of multiple words. On the other hand, text reading enables the evaluation of the syntactic advantage on the parafovea region.

## 5. Conclusions

In this study, we investigated the parafoveal semantic advantage netted by other linguistic factors that contribute to functional reading. Thus, future studies are required to further investigate how readers extract word meaning from the parafovea region during natural reading.

## Figures and Tables

**Table 1:** Mean values and standard deviations (in brackets) for the stimuli.

	Semantically Related (SR)		Semantically Unrelated (SU)	
	w1 Mean (SD)	w2 Mean (SD)	w1 Mean (SD)	w2 Mean (SD)
Short high-frequency words (*N* = 32)	length = 4.63 (0.50)	length = 4.63 (0.50)	length = 4.50 (0.52)	length = 4.38 (0.50)
	frequency = 2.47 (0.3)	frequency = 2.30 (0.4)	frequency = 2.40 (0.4)	frequency = 2.36 (0.4)
Short low-frequency words (*N* = 32)	length = 4.69 (0.47)	length = 4.63 (0.50)	length = 4.56 (0.51)	length = 4.50 (0.52)
	frequency = 0.89 (0.2)	frequency = 1.33 (0.6)	frequency = 0.98 (0.22)	frequency = 1.21 (0.5)
Long high-frequency words (*N* = 32)	length = 7.75 (0.86)	length = 7.88 (0.81)	length = 7.63 (0.72)	length = 7.94 (0.68)
	frequency = 2.02 (0.2)	frequency = 2.03 (0.2)	frequency = 2.07 (0.1)	frequency = 2.05 (0.2)
Long low-frequency words (*N* = 32)	length = 8.00 (0.82)	length = 7.63 (0.81)	length = 8.25 (0.77)	length = 7.81 (0.83)
	frequency = 0.87 (0.3)	frequency = 0.90 (0.3)	frequency = 0.77 (0.3)	frequency = 0.86 (0.3)

**Table 2:** Number and percentage of no responses and misread words in the different experimental conditions.

No Responses		Wilcoxon Test	Misread Words		Wilcoxon Test
short w2	long w2		short w2	long w2	
33.8% (*N* = 372)	66.2% (*N* = 729)	Z = 465; *p* < 0.001	60% (*N* = 282)	40% (*N* = 188)	Z = 40; *p* < 0.0001
low frequency w2	high frequency w2		low frequency w2	high frequency w2	
52.5% (*N* = 578)	47.5% (*N* = 523)	Z = 108; *p* = 0.052	49.6% (*N* = 233)	50.4% (*N* = 237)	Z = 146; *p* = 0.9
semantically related	semantically unrelated		semantically related	semantically unrelated	
42.1% (*N* = 464)	57.9% (*N* = 637)	Z = 40; *p* > 0.0001	42.8% (*N* = 201)	57.2% (*N* = 269)	Z = 50.5; *p* = 0.001

## Data Availability

The data that support the findings of this study are available upon reasonable request.
